# Exploratory feature analysis of baseline ionic currents in MoS_2_ nanopores

**DOI:** 10.1039/d6ra08707h

**Published:** 2026-10-07

**Authors:** Nicolas Vatiliotis, Azade YazdanYar, Ángel Díaz Carral, Maria Fyta

**Affiliations:** a Computational Biotechnology, Institute of Biotechnology, RWTH Aachen Worringerweg 52074 Aachen Germany mfyta@biotec.rwth-aachen.de; b Institute for Computational Physics, Universität Stuttgart Allmandring 3 70569 Stuttgart Germany; c Center for Computational Life Sciences, RWTH Aachen University Aachen Germany

## Abstract

Ionic-current fluctuations in nanometer-scale pores are relevant to hydrophobic gating in neuromorphic memristive systems and to signal interpretation in nanopore biosensing. While nanopore measurements commonly focus on transient current blockades, the statistical structure of baseline currents recorded in the absence of analytes has received less attention. Here, baseline recordings from two-dimensional MoS_2_ nanopores were analyzed to identify recurring signal descriptors associated with operationally assigned electrical regimes. Constant-voltage recordings were partitioned into analysis windows, from which statistical, temporal, and spectral descriptors were extracted and represented using kernel principal component analysis (Kernel PCA). Density-based clustering was used to identify outlying samples, while procrustes analysis ranked feature combinations according to how closely they preserved the geometry of the resulting embedding. Spectral descriptors were recurrent among the top-ranked features of the open-associated reference dataset, whereas the closed-associated reference dataset showed stronger contributions from temporal variability together with additional spectral descriptors. The mixed/transition-associated dataset exhibited combinations of descriptors observed across both reference regimes. A separate voltage-matched analysis of sequential closed-associated and open-associated sweeps from the same nanopore was included as a within-pore sensitivity analysis. This comparison held pore identity, nominal diameter, KCl concentration, and the applied-voltage set constant, although the sequential acquisition design does not exclude time- or order-dependent effects. Overall, baseline ionic-current recordings exhibited structured multivariate signatures that could be characterized through geometry-preserving feature analysis. This work proposes an exploratory analysis of the nanopore electrical-regime-associated signals without the need of direct data-intensive classification schemes.

## Abstract 2

Baseline ionic-current recordings in MoS_2_ nanopores show recurring spectral and temporal signatures strongly associated with nanopore electrical-regime-associated signals.

## Graphical abstract

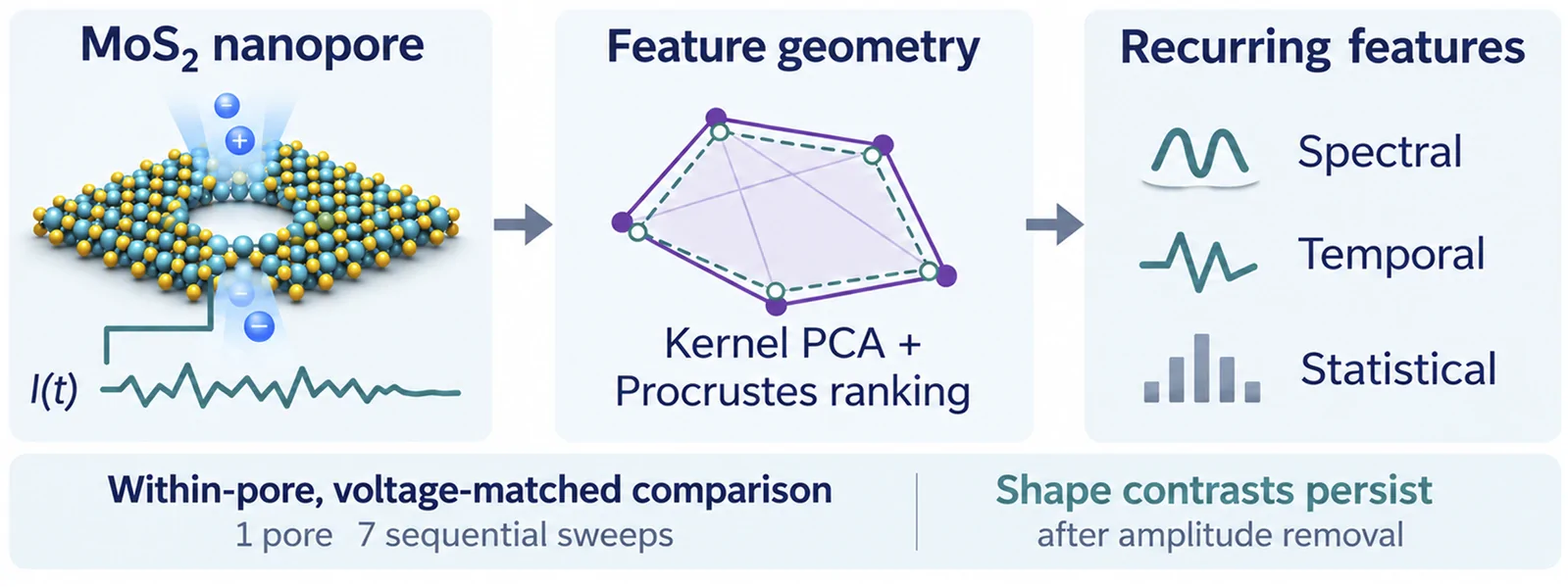

## 1. Introduction

The electrophoretic translocation of molecules through nanometer-sized pores has attracted attention over the last 30 years.^1–3^ Molecular translocation through a nanopore produces transient changes in the ionic current, allowing individual translocation events to be detected.^4,5^ This sensing principle underlies nanopore-based sequencing approaches.^3,6^ The driven polymer translocation through nanopores is a complex process involving electrohydrodynamics in the pore, as well as the non-equilibrium polymer dynamics originating from the fluctuations in the polymer conformations.^7,8^ The polymer-pore interactions and the role of the concentration gradient of the salt are important factors in the translocation of polyelectrolytes through nanopores.^9^ Additionally, the chain stiffness of semiflexible polyelectrolytes also plays an important role in controlling the translocation or threading time.^10^ Among all other factors, an important aspect is to control the translocation speed in order to efficiently probe the molecular internal composition with the range of temporal resolution and sensitivity available in experimental devices.

Motivated by these considerations, the present study shifts attention from individual molecular translocation events toward the statistical structure of the ionic current baseline. Previous studies have characterized ionic-current noise in solid-state nanopores, including low-frequency 1/*f* behavior and higher-frequency contributions associated with the electrical measurement system.^11^ These findings motivate examining the statistical structure of baseline-current recordings alongside their mean conductance. Wetting and hydration changes can influence ionic transport through nanopores, but similar changes in the recorded current may also arise from other pore-, experimental-, or measurement-related effects. A central interpretive difficulty is therefore distinguishing regime-associated changes in the current fluctuations from differences caused by experimental conditions or the measurement system. Electrical signals recorded from nanoscale devices often originate from physical processes that cannot be directly observed.^12^ In nanopore experiments, stochastic variations of the ionic current can reflect several underlying mechanisms, including changes in pore hydration, fluctuations of surface charge, or transitions between different transport regimes. Whether these fluctuations contain reliable information about the physical state of the nanopore, though, remains an open question. Beyond the relevance of a wet nanopore for biodetection, recently wetting was related to hydrophobic gating in bioinspired memristive nanopores.^13^

In view of these considerations, the present study examines whether statistical, temporal, and spectral descriptors of baseline-current segments exhibit recurring patterns associated with operationally assigned electrical regimes. Instead of focusing on translocation events, baseline segments recorded under constant voltage conditions are divided into analysis windows. For each window, a set of statistical, temporal, and spectral descriptors is computed. These descriptors include time-domain noise statistics together with parameters derived from power spectral density analysis, allowing a systematic characterization of the fluctuations present in the baseline current. In order to analyze how these descriptors are organized within the feature space, the resulting high-dimensional representation is mapped onto a nonlinear low-dimensional representation using kernel principal component analysis (Kernel PCA)^14^ in two (2D) and three (3D) dimensions. The embedding provides a geometric representation of the relationships between baseline noise descriptors. Density-based clustering (DBSCAN)^15^ is used to identify outlying samples in the two- and three-dimensional embeddings. The subsequent analysis identifies feature combinations that preserve the geometry of the resulting representations. The comparison of geometric configurations derived from multivariate datasets has a long tradition in statistics and shape analysis. Procrustes methods,^16^ as an example, were originally introduced to determine whether two point configurations share the same underlying structure after removing differences caused by translation, rotation, and scaling.^17,18^ These ideas were later extended to the simultaneous alignment of several configurations through generalised procrustes analysis,^19^ and the method has since become widely used in statistical shape analysis and geometric data analysis.^16,20^ Within this approach, the procrustes distance provides a measure of similarity between two point configurations that is independent of the coordinate system used to describe them.

In this work, procrustes analysis is used to compare coordinate representations derived from selected feature combinations with the reference geometry obtained from the Kernel PCA representations of the data in two and three dimensional spaces. During the alignment procedure, differences due to translation, rotation, and overall scaling are removed, allowing the comparison to focus only on the relative spatial relationships between points. The resulting procrustes disparity therefore indicates how closely a given feature combination reproduces the geometric organisation observed in the PCA projection or low-dimensional embeddings. When the disparity is low, the arrangement of points produced by the features closely follows the structure seen in the reference Kernel PCA. Feature sets that lead to smaller disparities therefore tend to preserve the internal organization of the baseline noise manifold. Such features summarize the statistical organization of the available recordings. Their association with an operational electrical regime does not, by itself though, establish a microscopic wetting state. In this work, we analyze data from nanopore experiments without the presence of analytes and provide a numerical scheme implementing the above methods. This approach provides a framework for characterizing baseline-current structure before analyte-translocation experiments and for identifying descriptors associated with operationally assigned electrical regimes. A separate voltage-matched comparison of sequential recordings from the same nanopore is included to assess these associations while holding pore identity and electrolyte concentration constant. The scope of our work is to identify considerable changes in the nanopore signals that could be indicative of changes in the electrical-regime-associated state without resorting to direct data-intensive classification schemes.

## 2. Methodology

### 2.1. Workflow

The main goal of this work is to examine whether statistical, temporal, and spectral descriptors of baseline ionic-current recordings contain structured signatures associated with operationally assigned nanopore regimes, and to identify feature combinations that preserve the geometry of the corresponding nonlinear representation. The computational workflow follows the steps summarized in Table 1. The following sections describe the individual stages of the analysis. In addition to the full analyzed signals, the final one-third and final one-quarter were examined to assess the sensitivity of the feature summaries to the analyzed signal portion. This exploratory analysis and comparisons evaluate within-recording consistency; it does not establish independent experimental reproducibility or wetting-state prediction accuracy.

**Table 1:** The distinct analysis steps, shown in the order provided by the numbers, included in the baseline-current feature-analysis workflow

Phase	Category	Description
(1) Pre-processing	Raw input	Ionic current, voltage, and conductance traces at a fixed sampling rate
	Windowing	Constant-voltage segmentation and generation of stationary sub-signals
(2) Feature extraction	TSFEL descriptors	Statistical, temporal, and spectral features of ionic-current fluctuations
(3) Feature refinement	Correlation filter	Removal of strongly correlated descriptors
	Variance filter	Removal of descriptors with negligible variance
	Standardisation	Column-wise *z*-score normalization
(4) Geometry analysis	Kernel PCA	Non-linear 2D and 3D embeddings of the feature space
	DBSCAN	Identification and removal of outlier samples
	Procrustes	Evaluation of feature combinations by alignment with the cleaned kernel PCA geometry

### 2.2. Experimental data

The ionic current recordings analyzed in this study originate from solid-state nanopore experiments through two-dimensional MoS_2_ pores performed in the Laboratory of Nanoscale Biology (LBEN) at the EPFL, Lausanne. In these, the voltage applied across the membrane was varied stepwise while the ionic current through the nanopore was monitored continuously. The resulting traces contain segments corresponding to different applied bias levels, each representing a stable voltage plateau within the stepped measurement protocol. The raw traces consist of segments recorded at multiple voltage levels rather than under constant-voltage conditions. Each raw trace contains three recorded quantities: ionic current, applied voltage, and conductance. The signals were sampled at a constant frequency of *f*_s_ = 100 kHz, producing uniformly spaced time-series data. No software filtering was applied to the ion-current channel, and the preamplifier had a bandwidth of 400 kHz. As the voltage changes occur during the measurements, the raw traces do not directly represent stationary baseline conditions and require segmentation prior to further analysis. The fourth dataset was used only for an exploratory comparison of its feature rankings with those obtained from the operationally assigned reference datasets. Its physical wetting state was not independently verified and therefore remains unknown. Note, that the assignment of the following experimental data to the electrical-regime-associated states was performed independently, after recording the data in the lab, and in advance to our analysis. Refer to the SI for additional details.^21^

The first three datasets were used as operational reference datasets for comparing baseline-current descriptors. Throughout this work, the terms “open-associated”, “closed-associated”, and “mixed/transition-associated” denote the electrical assignments used in the analysis. They do not constitute independent experimental verification of microscopic wetting states. The documentation available for this study does not establish that alternative explanations, including pore blockage, bubble formation, electrode malfunction, or contamination, were independently excluded. The fourth dataset was used only for an exploratory comparison with the reference feature summaries. Its physical wetting state was not independently verified and remains unknown. Because the original reference datasets differ in pore identity and experimental conditions, their feature differences cannot be attributed uniquely to wetting.

(1) ‘Open-associated’: a 3 nm nanopore in a 3 M KCl nanopore solution, with the applied voltage varying in the window [−200:−50] mV; the recording was assigned to the open-associated electrical regime; this assignment does not independently establish complete wetting.

(2) ‘Closed-associated’: a 10 nm nanopore in a 1 M KCl nanopore solution, with the applied voltage varying in the window [−700:−800] mV; the recording was assigned to the closed-associated electrical regime; low or unmeasurable ionic current does not, by itself, establish non-wetting.

(3) ‘Mixed/transition-associated’: a 3 nm nanopore in a 3 M KCl nanopore solution, with the applied voltage varying in the window [0:500] mV; the recording was assigned the operational label “mixed/transition-associated” from its electrical response. This assignment does not independently establish transitions between microscopic wetting states.

(4) ‘Unverified test dataset’: a 2.5 nm nanopore in a 3 M KCl nanopore solution, with the applied voltage varying in the window [−800:−700] mV; the physical wetting state was not independently verified and is therefore treated as unknown.

### 2.3. Within-pore, voltage-matched comparison dataset

An additional ‘control’ data set was analyzed separately to provide a within-pore, voltage-matched comparison for potential confounding by experimental conditions. The dataset comprises of seven consecutive voltage sweeps obtained from the same nominally 3 nm MoS_2_ nanopore in 3M KCl, using an identical set of applied voltages (0, ± 100, ± 200, ± 300, ± 400, and ± 500 mV). Based on the corresponding electrical response, IV1–IV3 were assigned the operational label “closed-associated”, whereas IV4–IV7 were assigned “open-associated”. These assignments were checked by direct inspection of the current–voltage records. No deliberate experimental intervention between IV3 and IV4 was reported in the information available to us. The labels therefore refer only to the observed electrical regimes and should not be interpreted as independent physical validation of the microscopic wetting state.

Both current and voltage channels were acquired at 100 kHz. An Axopatch filter was set to 10 kHz, no software filtering was applied to the ion-current channel, and the FEMTO preamplifier had a bandwidth of 400 kHz. Constant-voltage plateaus were partitioned into non-overlapping 2 s windows for subsequent analysis. Five duplicated −500 mV fragments with durations shorter than 2 s were retained in the data inventory, but excluded from feature extraction. In each affected sweep-voltage condition, however, a second plateau remained available and contained at least one complete 2 s window. For each analysis window, the mean current, current standard deviation, conductance at nonzero applied voltage, linear drift, distribution-shape statistics, and Welch power-spectral-density (PSD) descriptors were evaluated. PSDs were estimated using a Hann window with segments containing at most 16 384 samples, 50% overlap, constant detrending, and density scaling in A^2^ Hz^−1^. At a sampling rate of 100 kHz with 16 384-sample Welch segments, the frequency-bin spacing was approximately 6.10 Hz. Nominal band limits were evaluated using the available frequency bins.

Individual analysis windows were not considered as independent experimental replicates. Instead, window-level measurements, including values obtained from repeated plateau files at the same voltage, were first combined by taking the median within each sweep-voltage combination. For each matched voltage, regime-level summaries were then obtained as the median and interquartile range across the three closed-associated sweeps or the four open-associated sweeps. In the amplitude-ablation sensitivity analysis, each feature was robustly centered and scaled separately within each applied voltage before being summarized across the ten matched non-zero voltages for a given sweep. The scale-invariant feature set consisted of normalized PSD band fractions, PSD slope, lag-1 autocorrelation, skewness, and excess kurtosis. To verify numerically that these descriptors did not depend on the baseline-current offset or absolute fluctuation scale, feature extraction was repeated for the original signal, the mean-centered signal *I*(*t*) − *Ī*, and the standardized signal [*I*(*t*) − *Ī*]/*σ*_*I*_ in every valid window. Windows with zero or near-zero *σ*_*I*_ would have been excluded from standardization; none met this exclusion criterion. Because the seven sweeps represent sequential measurements of a single physical nanopore rather than independent experimental replicates, the comparison was restricted to features rank-biserial contrasts and leave-one-sweep-out ranges, and no inferential *p*-values were reported.

### 2.4. Segmentation and analysis windows

Raw recordings were segmented into intervals during which the applied voltage remained constant. These constant-voltage intervals provided the input for subsequent windowing and feature extraction. Constant applied voltage was not treated as proof of statistical stationarity. The full analyzed signals and their final one-third and one-quarter portions were compared to assess sensitivity to the analyzed signal portion. The shorter portions were not assumed to be intrinsically more stable or to provide independent observations.

In the reference segmentation implementation, the first and last 0.5 s of each constant-voltage trace are removed before division into complete, non-overlapping analysis windows; any incomplete remainder is omitted. The principal reference analyses used a nominal window duration of 2 s. The separate within-pore comparison used complete 2 s windows from the saved plateau files, without applying an additional 0.5 s boundary exclusion during that windowing step.

The 2 s segments were treated as temporal analysis windows rather than as independent experimental replicates. For assessments of reproducibility across devices, the relevant experimental unit is an independently fabricated and measured nanopore. Accordingly, multiple windows or repeated recordings obtained from the same pore were regarded as nested observations and were not counted as additional independent pores. The available reference datasets contain one named pore dataset for each operational regime; therefore, pore-level cross-validation and estimates of generalization to independently fabricated nanopores were not performed.

### 2.5. Feature extraction & refinement

Quantitative features were extracted from each analysis window using the Time Series Feature Extraction Library (TSFEL).^22,23^ We focus on features that capture the relevant statistical, temporal, and spectral properties of ionic current fluctuations. This controlled selection ensures consistent feature computation across datasets, while excluding features that are not meaningful and relevant to nanopore signals. The extracted variables describe statistical properties of the signal, temporal dynamics, and spectral characteristics of the ionic current fluctuations. In addition to the TSFEL-derived descriptors, two conductance-related quantities were computed directly from the signal: the mean conductance and the conductance standard deviation.

The spectral properties of the baseline noise were quantified through power spectral density (PSD) analysis. For each sub-signal, the PSD was computed and subsequently fitted within predefined frequency intervals using a power-law model. The fitting procedure yields scaling exponents describing the spectral behavior of the signal across frequency regimes. The estimated parameters, denoted *α*_0_ and *α*_1_, quantify the scaling of low-frequency and higher-frequency noise components, respectively. These spectral descriptors were appended to the feature representation of each sub-signal.

Prior to dimensionality reduction, the feature matrix was pre-processed in order to eliminate any redundancies and ensure numerical comparability across all descriptors. Pairwise Pearson correlation coefficients were computed for all feature pairs. For strongly correlated features, one was removed to reduce multi-collinearity. Features exhibiting negligible variance across samples were also discarded because such descriptors provide limited information power related to the electrical-regime-associated state identification. The remaining features were standardised through a column-wise *z*-score normalisation, ensuring comparable scaling across all feature dimensions through 
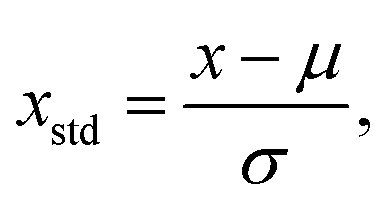
 where *x* denotes the original feature value, *µ* represents the mean of that feature across all samples, and *σ* corresponds to its standard deviation.

### 2.6. Nonlinear embedding and outlier detection

Non-linear dimensionality reduction was performed using kernel principal component analysis^14^ capturing non-linear relationships. We used a sigmoid kernel to construct the pairwise similarity matrix between samples. Two reduced representations were computed: a two-dimensional embedding (2D) and a three-dimensional embedding (3D). These embeddings represent a non-linear manifold capturing the low-dimensional geometric structure of the nanopore noise feature space. Density-based clustering with DBSCAN^15^ was applied independently on the 2D and 3D kernel PCA embeddings. The aim was to identify observations that deviate from the dominant low-dimensional structure and remove these. This filtering step produces a cleaned representation of the data that reflects the dominant geometric structure of the baseline characteristics and noise. The cleaned Kernel PCA embeddings provide thus the reference geometric configuration of the nanopore data.

### 2.7. Procrustes-based feature hierarchy

Procrustes analysis was used to evaluate how closely configurations constructed from selected descriptors could reproduce the geometry of the corresponding Kernel PCA embedding. All unordered feature pairs were considered for the 2D comparison, and all unordered feature triplets were considered for the 3D comparison. Each comparison used corresponding observations in the feature configuration and the outlier-filtered reference embedding. The alignment accounts for translation, rotation or reflection, and isotropic scaling. Procrustes disparity quantifies the residual geometric mismatch after alignment: smaller values indicate closer agreement between the configurations. Disparity is an outcome of this comparison, not a parameter that determines the embedding. The analysis ranks feature combinations according to their within-dataset geometric agreement. Neither low disparity nor frequent occurrence in selected combinations represents classification accuracy, a probability of wetting-state membership or predictive performance on an independently measured nanopore.

## 3. Results and discussion

### 3.1. Feature hierarchy

Applying the methodology described in Section 2 to the open-associated, closed-associated, and mixed/transition-associated reference datasets revealed a consistent set of conditions under which the geometric structure of the feature representations remains stable. The most reproducible configurations were obtained for window lengths of Δ*t* = 2 s and for a procrustes disparity value around *d* = 0.2. This procrustes disparity value ensured the same parameters across the datasets. From all feature combinations, the top 10 features with the smallest procrustes disparity values were selected for each of the sets, and their frequency in the set was then evaluated. These observations therefore mark the parameter regime in which the geometric relationships between the datasets become most clearly expressed. The definitions and physical meanings of extracted features are interpreted using the standard classification used in time-series analysis, which classifies the feature groups on the signal representation they come from. The occurrence frequency used in the following analysis refers to the number of times a given feature appears among the ten feature combinations with the smallest procrustes disparity values. It therefore reflects the reoccurrence of a feature within the best-performing procrustes disparity values. In this sense, the occurrence frequency serves as a measure of feature consistency within the low-disparity set.

### 3.2. Within-pore comparison under matched experimental conditions

The within-pore comparison dataset included 84 constant-voltage plateau files, of which 79 yielded 239 non-overlapping 2 s analysis windows. Five short duplicate −500 mV fragments were excluded without removing any required sweep-voltage combination. After aggregation, each matched voltage contained three closed-associated sweeps (IV1–IV3) and four open-associated sweeps (IV4–IV7). The matched current–voltage curves showed a substantially larger absolute current in the open-associated sweeps across the nonzero voltage range, and their current-noise magnitude was generally larger (Fig. 1). At +500 mV, the median current was 935 pA (0.94 nA) in the closed-associated regime and 73 674 pA (73.7 nA) in the open-associated regime; the corresponding median conductances were 1.87 and 147.35 nS.

**Fig. 1:**
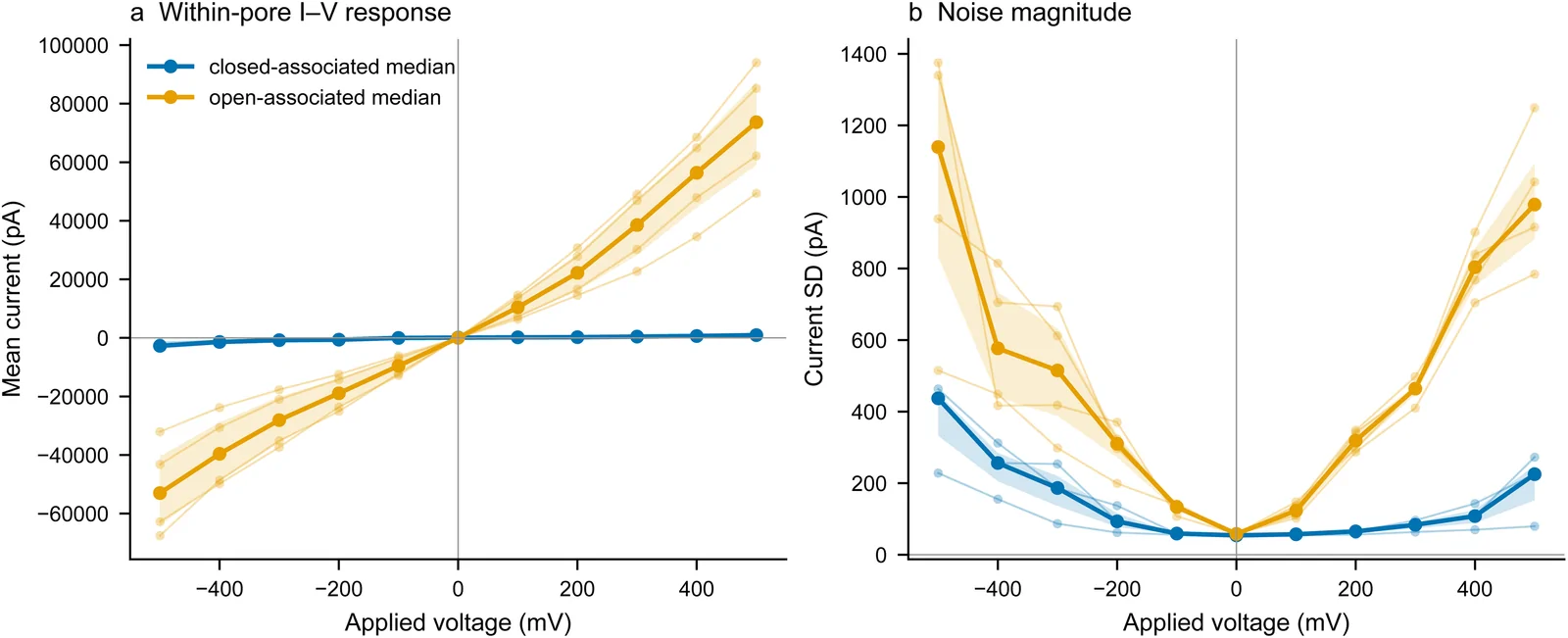
Occurrence frequencies of the ten top-ranked features from each of the closed-associated and open-associated datasets, using the full analyzed signals. The combined feature lists are shown on the *x*-axis. Bold *x*-axis labels indicate features appearing in the displayed top-ten list of only one dataset; regular-weight labels indicate features shared by both lists. Red numbers indicate the associated procrustes disparity values, which quantify geometric agreement rather than prediction accuracy.

After current amplitude, conductance, and absolute noise-magnitude descriptors were excluded, six of seven shape-only descriptors had an absolute descriptive rank-biserial contrast of 1.0, with an unchanged sign in every leave-one-sweep-out calculation (Fig. 2). These descriptors were the three normalized PSD band fractions, the PSD slope from 10–1000 Hz, lag-1 autocorrelation, and excess kurtosis. Skewness was weaker (−0.25), and its leave-one-sweep-out range included zero (−0.667 to 0). Representative current–time traces and physical-unit PSDs from the adjacent IV3 and IV4 sweeps are provided in the SI.

**Fig. 2:**
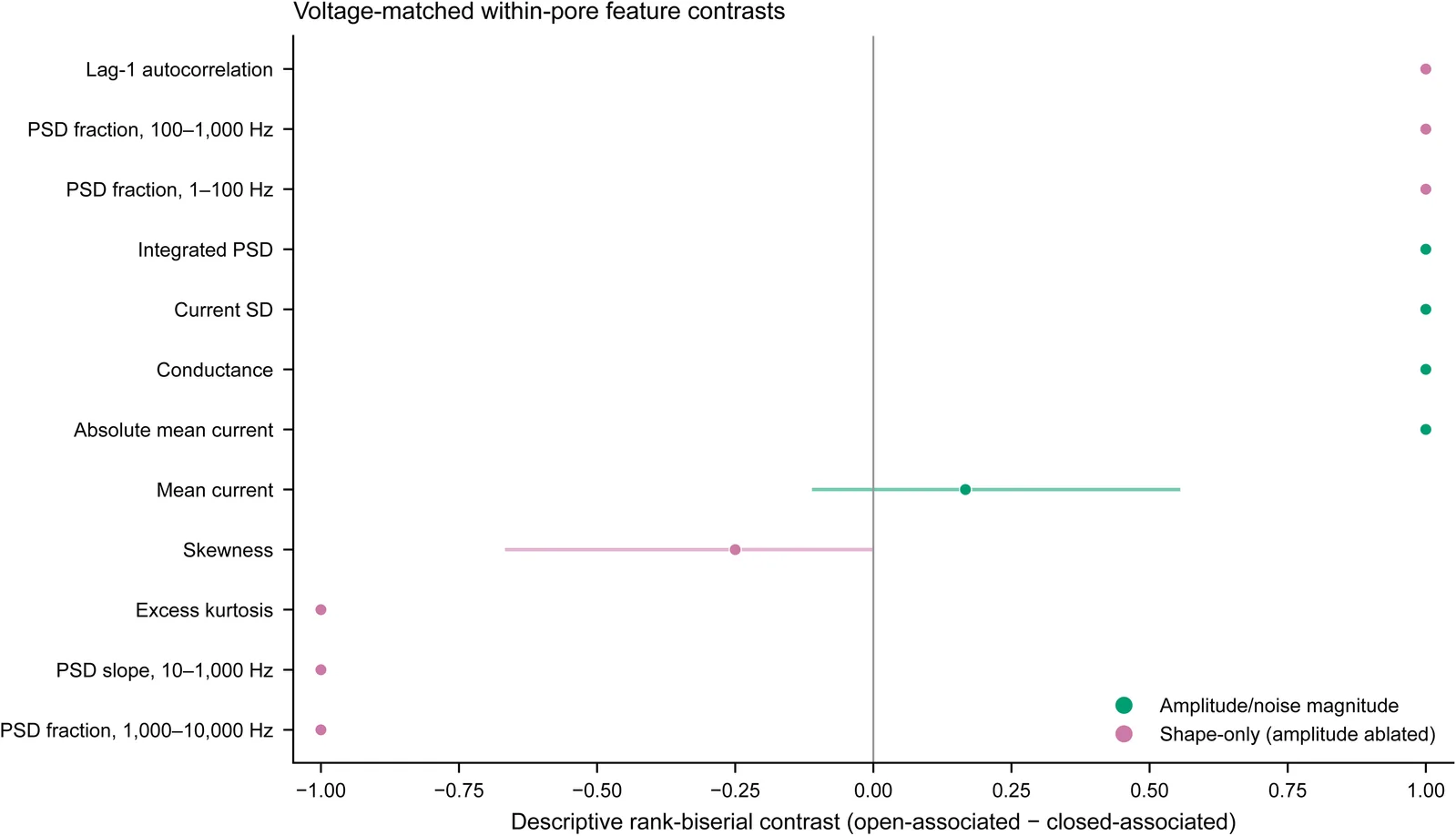
Voltage-matched electrical comparison for the within-pore comparison dataset. (a) Mean current as a function of applied voltage for IV1–IV3 (closed-associated) and IV4–IV7 (open-associated). Thin lines denote individual sweeps; thick lines and shaded regions denote the regime median and interquartile range across sweeps. (b) Current standard deviation at the same matched voltages. Two-second analysis windows and repeated plateaus were aggregated within each sweep-voltage combination before plotting. All seven sweeps were obtained from one physical pore; the shaded regions do not represent independent-pore confidence intervals.

The scale-invariant descriptors were also recalculated from mean-centered and standardized versions of all 239 valid windows. All seven descriptors agreed with their original-signal values within the predefined numerical tolerance under both transformations (14 of 14 descriptor-transformation checks; SI Section 2), with no near-zero-variance exclusions. The corresponding descriptive regime contrasts are reported in SI Table S1. These checks verify that the implemented descriptors are numerically insensitive to the baseline-current offset and absolute fluctuation scale. Such agreement is expected for appropriately defined scale-invariant descriptors and is not an independent validation of feature selection. The reported contrasts therefore persist in descriptors constructed to exclude absolute current and fluctuation magnitude. They remain descriptive observations from one physical pore.

These observations can be considered alongside previous investigations of nanopore current noise. Smeets *et al.*^11^ reported substantial differences in low-frequency noise between nanopores of similar diameter, examined its dependence on electrolyte concentration, and modeled higher-frequency noise using the electrical measurement circuit. Their findings illustrate why spectral differences must be interpreted in relation to both pore characteristics and the measurement system. In our comparison within the ‘control’ data, the pore identity, electrolyte concentration, and applied voltage were matched, and descriptor contrasts remained after mean centering and amplitude standardization. However, these transformations do not isolate a particular noise source: differences in the relative contributions of pore fluctuations and instrumental noise could still affect the normalized spectral shape.

Hydrophobic gating in memristive nanopores^13^ provides a relevant physical context for considering wetting-related changes in ionic transport. The electrical-regime differences observed here do not establish that the same mechanism operated in these recordings. We therefore interpret the descriptor patterns as associations with the observed electrical regimes, while leaving their microscopic origin unresolved. A descriptive sensitivity analysis of all six possible boundaries between consecutive sweeps did not uniquely identify the operational IV3|IV4 division. Both the adjacent-sweep separation and the balanced between-segment separation were largest at IV2|IV3; IV3|IV4 ranked third under both measures (SI Table S2). Thus, the group-level descriptor contrasts do not establish that the strongest multivariate change coincided with the operational regime boundary. This matched comparison demonstrates a change in operational electrical regime in one pore under the same named experimental conditions. It does not establish that wetting was the only microscopic property that changed. The two regimes were measured sequentially; their physical wetting states were not independently validated in the available records, and time-dependent pore evolution remains a possible explanation.

### 3.3. Full signal analysis

We begin the analysis with assessing the relevance of the features in the operationally assigned reference datasets, open-associated and closed-associated reference datasets. The influence of the most relevant features according to the workflow described above can be observed in Fig. 3. In this, the occurrence frequency of the individual features that appear among the ten feature combinations with the smallest procrustes disparity values is shown. Interestingly, for both reference datasets, spectral and temporal features occur most frequently with the spectral domain being favored even more than the temporal one. This indicates that frequency-domain descriptors are prominent in the selected feature combinations for both reference datasets.^24,25^ Features such as entropy, spectral entropy, spectral kurtosis, maximum frequency, power bandwidth, and absolute energy shared between both reference datasets, describe signal properties that remain relevant in both pore conditions. At the same time, though it is evident from the figure that distinct descriptors show different recurrence patterns between the two reference datasets. These are the neighborhood peaks, spectral distance, mean absolute difference, and negative turning points that are more frequent in the closed-associated reference dataset. These descriptors concern local fluctuations and spectral structure; their occurrence counts do not establish larger fluctuation magnitudes or greater spectral complexity. In the open-associated spectral decrease, spectral skewness, wavelet entropy, spectral centroid, and spectral variation have a higher occurrence indicating a stronger contribution from features related to spectral shape, asymmetry, and energy distribution.^24^ The full-signal analysis therefore identifies recurring feature combinations within the reference datasets, rather than an independently validated single-feature marker of wetting state.

**Fig. 3:**
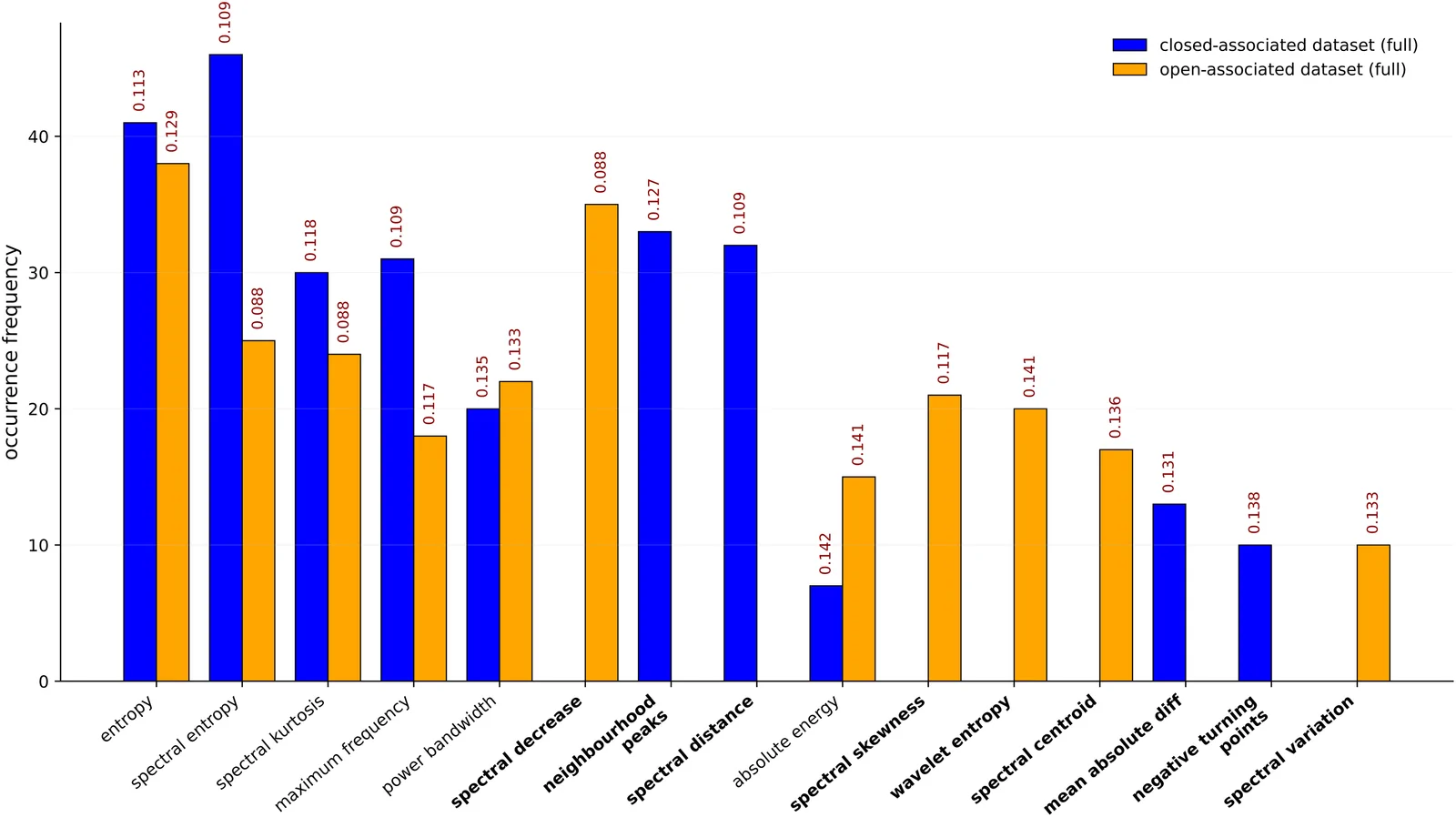
Voltage-matched feature sensitivity after amplitude ablation. Descriptive rank-biserial contrasts compare open-associated and closed-associated sweeps after robust within-voltage standardization. Points show the complete seven-sweep contrast, and horizontal lines show the range obtained after omitting one sweep at a time. Shape-only descriptors exclude current amplitude, conductance, and absolute noise magnitude. These contrasts describe within-pore stability and are not independent-pore effect estimates, inferential tests, or prediction metrics.

### 3.4. Features in the final 1/3 signal segment

In order to assess the conservation of the representative features from the full signals, we repeat the analysis with the final one third segment of the signal for the same open-associated and closed-associated reference datasets. The results are depicted in Fig. 4. Again, temporal and mainly spectral features dominate with the feature conductances appearing here. The shared features (maximum frequency, absolute energy, spectral kurtosis, negative turning points, spectral centroid) in both reference datasets are similar but not identical as for the full signal. Clear differences can be observed in features that occur preferentially in one of the reference datasets. For the closed-associated reference dataset, there are spectral distance, power bandwidth, spectral entropy, neighborhood peaks, mean absolute diff, and conductances. For the open-associated reference dataset, entropy, spectral decrease, wavelet entropy, median frequency, and spectral skewness. Even for this reduced signal segment, the different characteristics of the open-associated and closed-associated reference datasets are mapped onto distinct features.

**Fig. 4:**
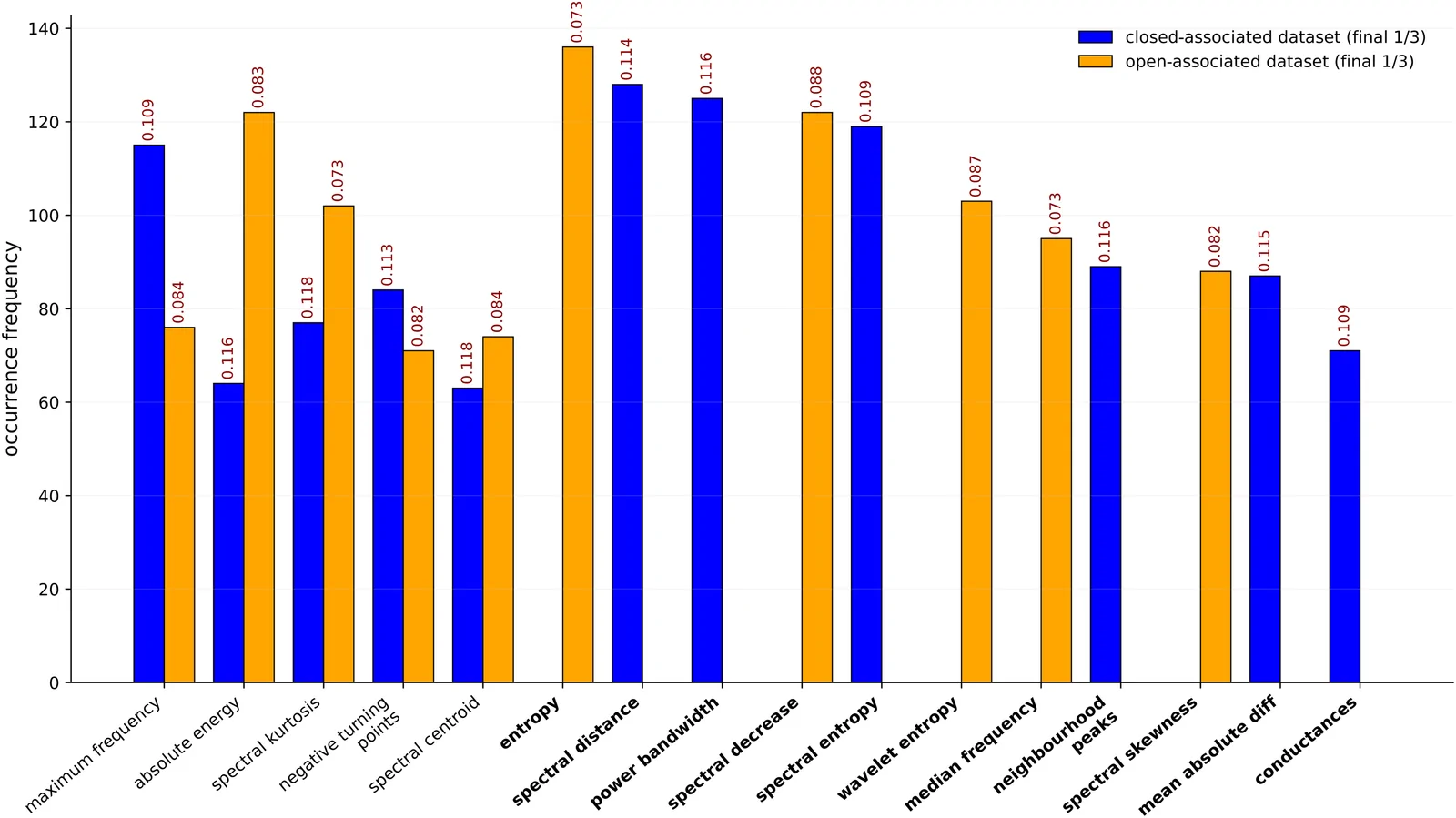
Occurrence frequencies of the ten top-ranked features from each of the closed-associated and open-associated datasets, using the final third of each analyzed signal. The combined feature lists are shown on the *x*-axis. Bold *x*-axis labels indicate features appearing in the displayed top-ten list of only one dataset; regular-weight labels indicate features shared by both lists. Red numbers indicate the associated procrustes disparity values, which quantify geometric agreement rather than prediction accuracy.

A comparison of Fig. 3 and 4 reveals that the main spectral structure is preserved both in the full and the reduced signals. In both analyses, spectral descriptors remain prominent among the recurring features. This demonstrates recurrence within a later portion of the same recordings, rather than independent experimental validation of regime discrimination across nanopores. At the same time, the reduction of the signal window modifies the recurrence pattern of individual features. The main difference between the two signal lengths refers to the type of information that is prominent in the closed-associated reference dataset. While neighborhood peaks, spectral distance, and mean absolute differences remain frequent in both segments, the final 1/3 segment enhances the meaning of power bandwidth, spectral entropy, and conductances, including descriptors of spectral complexity, bandwidth, and electrical response in the closed-associated dataset. The full signal reveals a relatively stronger contribution from negative turning points, preserving contributions from local time-domain fluctuations. In the open-associated regime, the final 1/3 segment enhances the contribution of median frequency and entropy; the latter not occurring in the closed data comparing to the full signal for which spectral centroid was more prominent and not occurring in the final 1/3 segment. The results suggest that the final signal segment preserves the main regime-associated organisation but shifts the relative importance of specific descriptors. Accordingly, the full and final 1/3 signals retain consistent, but not identical information. In fact, the reduced signal window emphasizes features associated with the later spectral organisation of the current fluctuations.

### 3.5. Features in the final 1/4 signal segment

In order to support our observations, we perform one more analysis with the final quarter of the signal summarized in Fig. 5. Inspection of the depicted results reveals that the displayed reference feature lists again contain descriptors from the frequency and time domains. The features entropy, spectral entropy, negative turning points, mean absolute difference, and spectral centroid are shared between the two displayed reference lists. For this shorter segment the open-associated regime is dominated by features of the spectral domain (spectral decrease, wavelet entropy, power bandwidth, median frequency, and spectral skewness), while the closed-associated regime is dominated by features of both the spectral and temporal domain (maximum frequency, neighborhood peaks, spectral kurtosis, conductances, absolute energy, and skewness). Accordingly, the open-associated reference dataset is more strongly associated with spectral shape, spectral complexity, and energy distribution across frequencies and scales, while the closed-associated reference dataset retains a stronger contribution from local fluctuation, amplitude, and distribution-based features. In the final 1/4 segment, the open-associated reference dataset enhances the occurrence frequency of several features, is thus represented by a more concentrated set of strongly recurring features. The closed-associated reference dataset is described by a broader set of features with lower occurrence counts, being represented by a more distributed recurrence pattern across several features. A comparison of Fig. 3 and 5 reveals the preservation of the main regime-associated spectral and temporal characteristics, indicating that the differences between the open-associated and closed-associated feature profiles do not arise primarily from the early portion of the analyzed recordings.

**Fig. 5:**
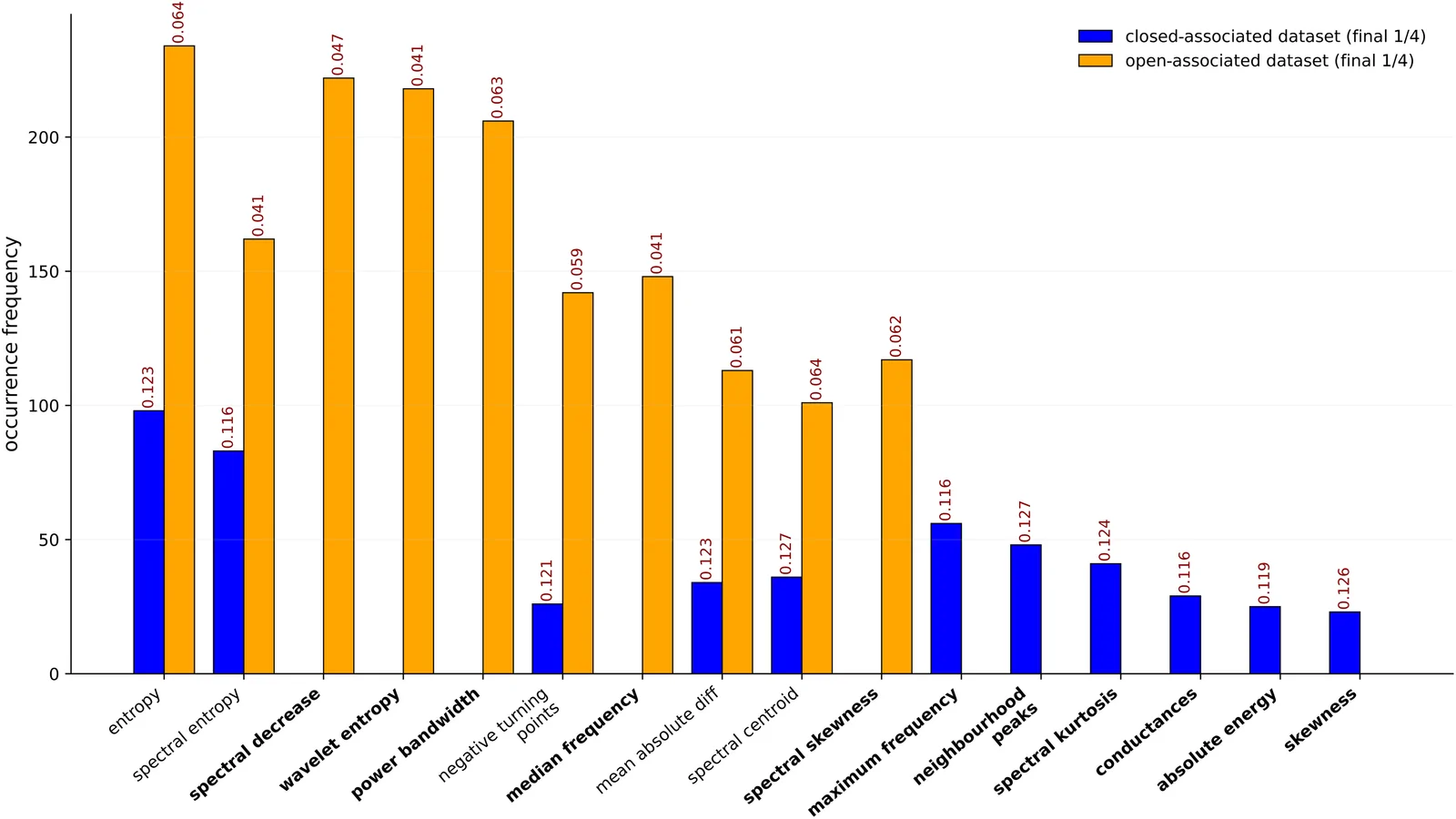
Occurrence frequencies of the ten top-ranked features from each of the closed-associated and open-associated datasets, using the final quarter of each analyzed signal. The combined feature lists are shown on the *x*-axis. Bold *x*-axis labels indicate features appearing in the displayed top-ten list of only one dataset; regular-weight labels indicate features shared by both lists. Red numbers indicate the associated procrustes disparity values, which quantify geometric agreement rather than prediction accuracy.

We next compare the most relevant features obtained for the full signal and the final 1/4 segment. For the closed dataset, the 1/4 segment enhances the contribution of features related to amplitude distribution and signal shape. Still it preserves the observation from the full signal analysis that the closed-associated regime is dominated by features related to temporal and spectral structure remains. For the open dataset the same comparison supported the finding that the open-associated regime is dominated by features primarily related to spectral domain. Note though that some features such as power bandwidth are found only in the open-associated regime in the 1/4 segment analysis, while for the full signal this was found in both open and closed datasets. An overall comparison of Fig. 4 and 5 underlines the fact that the open-associated regime remains strongly associated with spectral decrease, wavelet entropy, and other high-recurrence spectral features, while the closed-associated regime retains recurrent contributions from features linked to local fluctuations and signal variability. A comparison of the 1/4 to the 1/3 segment emerged features reveals that in the open-associated regime the former preserves the strong contribution of spectral decrease and wavelet entropy, while power bandwidth, median frequency, and spectral skewness remain prominent. The 1/4 segment, in the closed-associated regime, emphasizes maximum frequency, apectral kurtosis, absolute energy, and skewness over spectral distance, power bandwidth, and spectral entropy in the 1/3 analysis. Accordingly, compared to the 1/3 segment, the 1/4 segment still retains the essential regime-associated information shifting the relative importance of the features towards a narrower and more strongly recurring subset in the open-associated regime and a broader moderate-frequency set in the closed-associated regime.

### 3.6. Recurring dataset-associated descriptors

A joint evaluation of the analysis of all three signal segments (full, final 1/3, final 1/4) enable us to propose dataset-associated descriptors. These were classified with respect to their appearance consistency in the analyses. Features that remained exclusive to one dataset's displayed list in all three analyses were classified as ‘main’; if these remained electrical-regime-associated specific in two of the three analyzed signal segments they were classified as ‘secondary’ electrical-regime-associated characteristic features as summarized in Table 2. Evidently spectral decrease, spectral skewness, and wavelet entropy are descriptors exclusive to the displayed open-associated lists, while only neighborhood peaks is exclusive to the displayed closed-associated lists. Regarding the secondary specific features only median frequency is open-associated reference dataset specific, while spectral distance, mean absolute difference, and conductances are classified as secondary characteristic of the closed-associated regime. Once all pore-specific features that are either shared or found in the other electrical-regime-associated state or in any other signal segment are excluded one descriptor per reference dataset remains under this list-membership criterion as shown in Table 3. The listed features are unique to one electrical-regime-associated state, appear in only one signal segment, and do not occur as shared features in the other analyses are thus electrical-regime-associated throughout our analyses. For the open-associated reference dataset state, spectral variation remains unique only in the full-signal analysis. For the closed-associated reference dataset state, skewness remains unique only in the last one-quarter analysis. This strong reduction of the electrical-regime-associated specific feature list under this stricter criterion indicates that most features identified in the different signal segments are either preserved across multiple segments or lose their electrical-regime-associated specificity during the comparison. Accordingly, the most stable pore-specific characteristics are mapped mainly onto the main and secondary features (Table 2), whereas only single features are strictly unique for a single signal segment (Table 3).

**Table 2:** Recurring dataset-associated descriptors across the full-signal, final-third, and final-quarter analyses. “Main” denotes exclusive membership in one dataset's displayed list in all three analyses; “secondary” denotes exclusive membership in two analyses

Reference dataset	Class	Features
Open-associated	Main	Spectral decrease, spectral skewness, wavelet entropy
Open-associated	Secondary	Median frequency
Closed-associated	Main	Neighborhood peaks
Closed-associated	Secondary	Spectral distance, mean absolute diff, conductances

**Table 3:** Descriptors appearing exclusively in one dataset and one signal-portion list within the displayed reference comparison s. Exclusivity refers only to these feature lists and does not establish a unique physical-state marker

Reference dataset	Signal segment	Features
Open-associated	Full	Spectral variation
Closed-associated	Final 1/4	Skewness

### 3.7. Mixed/transition-associated dataset

We next focus on the mixed dataset, which exhibits a more heterogeneous geometric organisation than the open or closed data, with the aim to assess whether the mixed/transition-associated dataset retains descriptors identified in the open-associated and closed-associated reference datasets discussed above. For this, descriptors observed in both operational reference datasets appear within the same recording, showing overlap between the feature lists of the operational reference datasets. This is depicted in Table 4, which summarizes the frequently occurring features in the mixed dataset, color to annotate of the feature specificity (in the open, closed or mixed data) and does not modify the procrustes ranking itself. Inspection of the listed features reveals that the full signal analysis of the mixed data retains contributions from both electrical-regime-associated specific descriptors. When the analysis is restricted to the final 1/3 segment, the mixed/transition-associated dataset retains closed-state specific features, namely mean absolute difference. Additional features such as slope, spectral spread, and centroid appear in the mixed data ranking that were not open/closed-associated reference dataset specific. The frequent occurrence of these new features suggests that the reduced signal segment captures signal properties that are not fully represented in the two operational reference datasets. The displayed mixed/transition-associated feature lists therefore contain both overlapping and additional descriptors relative to the other reference lists. Their microscopic physical interpretation remains unresolved.

**Table 4:** Top-ranked features obtained for the mixed dataset from the 3D analysis of the full signal, the last 1/3, and the last 1/4 segment of the signal. For each signal segment, the feature, the corresponding procrustes disparity value (DV), and the occurrence frequency (occ.freq.) are displayed. The features are color-coded according to their relevance to the operational descriptor groups: red, open-associated; blue, closed-associated; green, mixed/transition-associated; and black, shared or unassigned

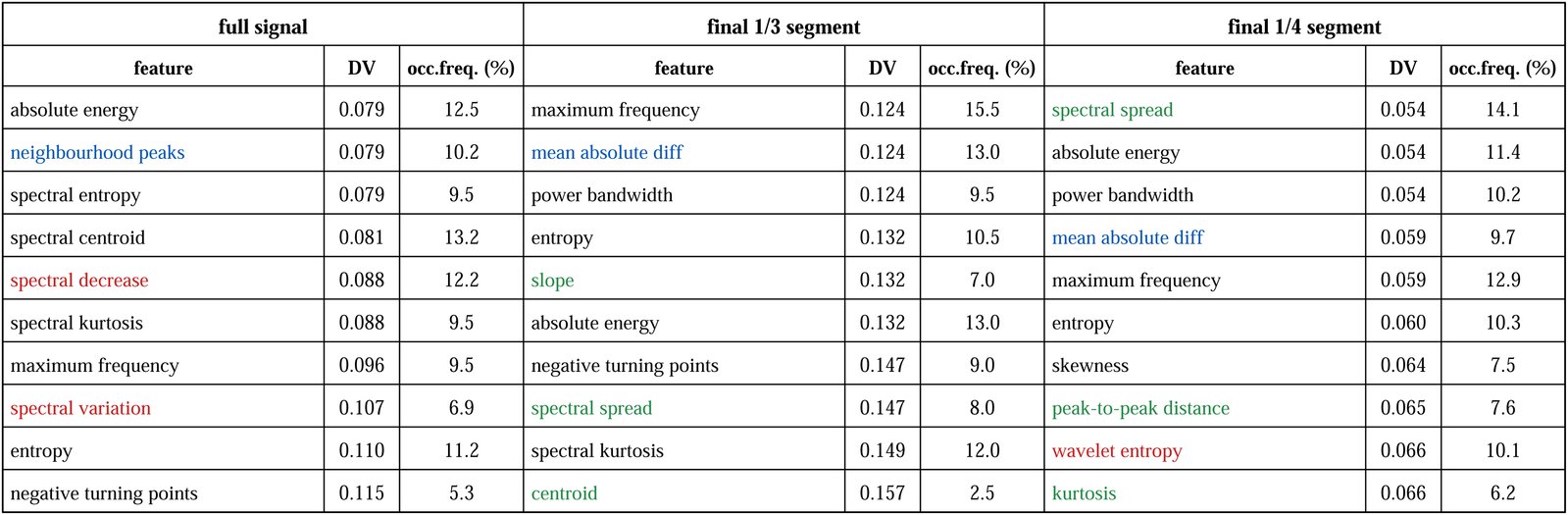

The final-third list does not include the descriptors designated as main open-associated descriptors in Table 2. This is a comparison of list membership, and should not be interpreted as evidence for a particular microscopic pore configuration. Interestingly, the final 1/4 segment re-enhances the open-associated reference dataset characteristics as wavelet entropy reappears. At the same time the mixed/transition-associated dataset characteristics feature peak-to-peak distance is particularly relevant as it reflects the spacing between local extrema in the time trace not captured within the open- and closed-pore current traces. Intuitively, the occurrence of spectral spread indicates a broadening of the spectral distribution, while kurtosis points to changes in the shape of the distribution of signal amplitudes. Taken together, the analysis of the mixed/transition-associated dataset supports a comparative interpretation of the feature space of the feature space. The open-associated and closed-associated reference datasets are mainly described by stable spectral-shape features and features related to local fluctuations and signal variability, respectively. The mixed/transition-associated dataset includes such features but enhances the occurrence of features, such as spectral spread, slope, centroid, peak-to-peak distance, and kurtosis, not observed for the distinct operational reference regimes. The mixed/transition-associated dataset therefore contains additional baseline-current descriptors that were not prominent in the two operational reference datasets. The microscopic origin of these differences cannot be resolved from the available recordings. Since nanopore noise is strongly reflected in spectral and time domains, changes in wetting or partial obstruction are expected to alter not only the average conductance but also the organisation of fluctuations in the signal.^24,25^ The mixed/transition-associated dataset therefore exhibited a distinct multivariate feature profile, containing descriptors observed in both operational reference datasets together with additional recurring descriptors. This overlap is descriptive and does not establish an intermediate microscopic wetting state.

### 3.8. Exploratory comparison with an unverified test dataset

We next applied the same feature-ranking workflow to the unverified test dataset, for which the physical wetting state was not independently verified. The purpose was limited to comparing its frequently ranked descriptors with those observed in the operationally assigned reference datasets. This comparison is exploratory; it is not a blinded classification test and does not provide an estimate of predictive performance. The resulting feature rankings are presented in Table 5. Across the full signal, final one-third, and final one-quarter analyses, the test dataset contained combinations of descriptors previously observed in the open-associated, closed-associated, and mixed/transition-associated reference datasets. For example, the final one-third contained spectral decrease and spectral distance, previously associated with different operational reference regimes, together with centroid and slope. The final one-quarter additionally contained peak-to-peak distance, which occurred among the descriptors of the mixed/transition-associated reference dataset. These observations establish only a descriptive similarity between the feature composition of the unverified test dataset and those of the operational reference datasets. They do not demonstrate that the test pore was physically in a mixed or partially wet state. Because no independent experimental validation is available, the correctness of any wetting-state assignment cannot be evaluated from these data.

**Table 5:** Top-ranked feature summary for the unverified test dataset from the three-dimensional analysis of the full signal, final third, and final quarter. DV denotes the reported procrustes disparity value, and occ.freq. denotes the reported occurrence percentage. Color annotations provide a descriptive cross-reference to the operational descriptor groups: red, open-associated; blue, closed-associated; green, mixed/transition-associated; and black, shared or unassigned. Descriptor overlap does not constitute a validated classification of the test pore's physical wetting state

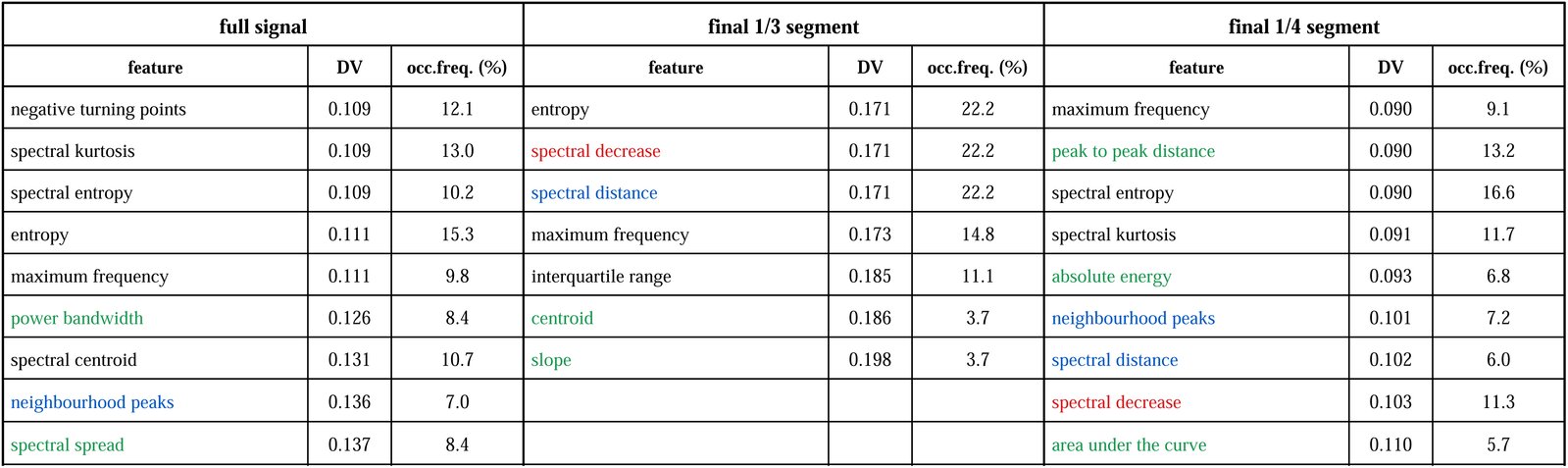

## 4. Conclusions

The combination of time-series feature extraction, non-linear embedding, and procrustes-based geometric comparison applied in this work to ionic current traces from solid-state nanopore experiments enabled the systematic identification of recurring descriptors associated with operationally assigned electrical regimes. These are characterized as open-associated, closed-associated, and mixed/transition-associated reference datasets, without implying independent validation of their microscopic wetting states. Using a window length of Δ*t* = 2 s together with a 3D kernel PCA representation and the selected procrustes disparity range, we examined the recurrence of descriptors within feature combinations that preserved the geometry of the nonlinear embedding. Key in the proposed workflow is the procrustes analysis used to identify the feature combinations that best retained the characteristics of the reference non-linear geometric representation.

The comparison across the full signal, the final 1/3, and the final 1/4 signa segments revealed that several descriptors recurred across these analyses, although the feature lists were not identical. The different signal portions therefore provided complementary summaries of the baseline-current structure. This is especially important for the mixed/transition-associated dataset, where different signal segments emphasized different combinations of descriptors, including features shared with the open-associated and closed-associated reference datasets. The full signal provides a summary over the complete analyzed recording, while the reduced signal segments are useful for examining the sensitivity of the feature lists to the analyzed signal portion.

Importantly, our analysis identified recurring feature combinations within the available datasets rather than a single validated wetting-state marker. The open-associated reference dataset retained recurrent spectral descriptors relevant primarily to the organisation of the signal in the frequency domain, while the closed-associated reference dataset also retained descriptors of signal variability and local fluctuation. The mixed/transition-associated reference dataset contained descriptors observed in both other reference datasets, together with additional descriptors. These differences concern the composition of the selected feature lists; they do not establish physical switching between wet, non-wet, or partially obstructed configurations. The exploratory comparison showed that the unverified test dataset shared frequently ranked descriptors with the operational reference datasets, including descriptors associated with the mixed/transition-associated regime. Because the physical state of the test dataset was not independently verified and independent pore-level validation was unavailable, this similarity cannot be interpreted as a validated prediction of its wetting state.

The within-pore comparison compared sequential recordings from the same nanopore at matched applied voltages, with the same nominal pore diameter and KCl concentration. This reduced confounding by these factors within the comparison dataset. The selected shape descriptors and their regime contrasts were numerically unchanged after mean centering and amplitude standardization. However, the strongest descriptive separation between consecutive sweeps occurred at IV2|IV3 rather than at the operational IV3|IV4 boundary. Note that IV2 and IV3 both belong to the closed-associated group, meaning that the largest descriptor change occurred within the same operational regime rather than at the assigned boundary between IV3 and IV4. The group comparison and the boundary analysis thus address different aspects of the data. The group comparison summarizes the overall differences between the assigned regimes, whereas the boundary analysis probes the strongest change along the sequence of measurements. Accordingly, the observed group-level differences are not necessarily linked to descriptors uniquely identifying the transition between IV3 and IV4. The descriptor variation within the closed-associated group rather reflects time-dependent pore changes, recording drift, or other sources of variation.

The comparison comprises sequential measurements from one physical pore and does not independently validate microscopic wetting states. The observed differences describe a change in electrical behavior under the specified matched conditions, but cannot identify wetting as the only cause, exclude time- or order-dependent changes, or demonstrate generalization across independently fabricated pores. Overall, this work investigated whether baseline ionic current fluctuations contain structured signatures associated with operational electrical regimes in solid-state nanopores. The proposed analysis workflow and the gained insights provide an exploratory framework for characterizing these signatures through geometric feature comparison. Our analysis approach may support the interpretation of baseline electrical signals before ionic and molecular gating experiments, alongside conventional current and conductance summaries, without the need of classifying directly the electrical-regime-associated signal. Such characterization is relevant to nanopore biosensing and to the study of electrical behavior in bioinspired neuromorphic systems. Independent physical-state validation and measurements from multiple independently fabricated nanopores are required before the descriptors can be used to establish regime-associated or predictive generalization.

## Conflicts of interest

The authors have no conflicts to disclose.

## Supplementary Material

RA-016-D6RA08707H-s001

## Data Availability

The data that support the findings of this study are available from the corresponding author upon reasonable request. For inquiries on the experimental procedures contact to the relevant laboratory will be provided upon reasonable request. The code that was used is available under https://github.comRWTH-CompBioTech/nanopore_noise_analysis. Supplementary information (SI) is available. See DOI: https://doi.org/10.1039/d6ra08707h.
