# Supplementary material for: Exploratory feature analysis of baseline ionic currents in MoS2 nanopores
Source: RSC Adv. 2026 Oct 7;16(46):60584–96. doi: 10.1039/d6ra08707h (PMC13643522; doi:10.1039/d6ra08707h)
Supplement: Supplementary file 1 [file RA-016-D6RA08707H-s001.pdf]

Supplementary Information (SI) for RSC Advances.
This journal is © The Royal Society of Chemistry

Supplementary Information: Exploratory feature analysis of baseline ionic currents in MoS_2 nanopores

Nicolas Vatiliotis,[1] Azade YazdanYar,[2] Angel Diaz Carral,[2] and Maria Fyta[1,3]

[1] *Computational Biotechnology, Institute of Biotechnology, RWTH Aachen, Worringerweg, 52074 Aachen, Germany*

[2] *Institute for Computational Physics, Universität Stuttgart, Allmandring 3, 70569 Stuttgart, Germany*

[3] *Center for Computational Life Sciences, RWTH Aachen University, Aachen, Germany*[a]

[a] Electronic mail: mfyta@biotec.rwth-aachen.de

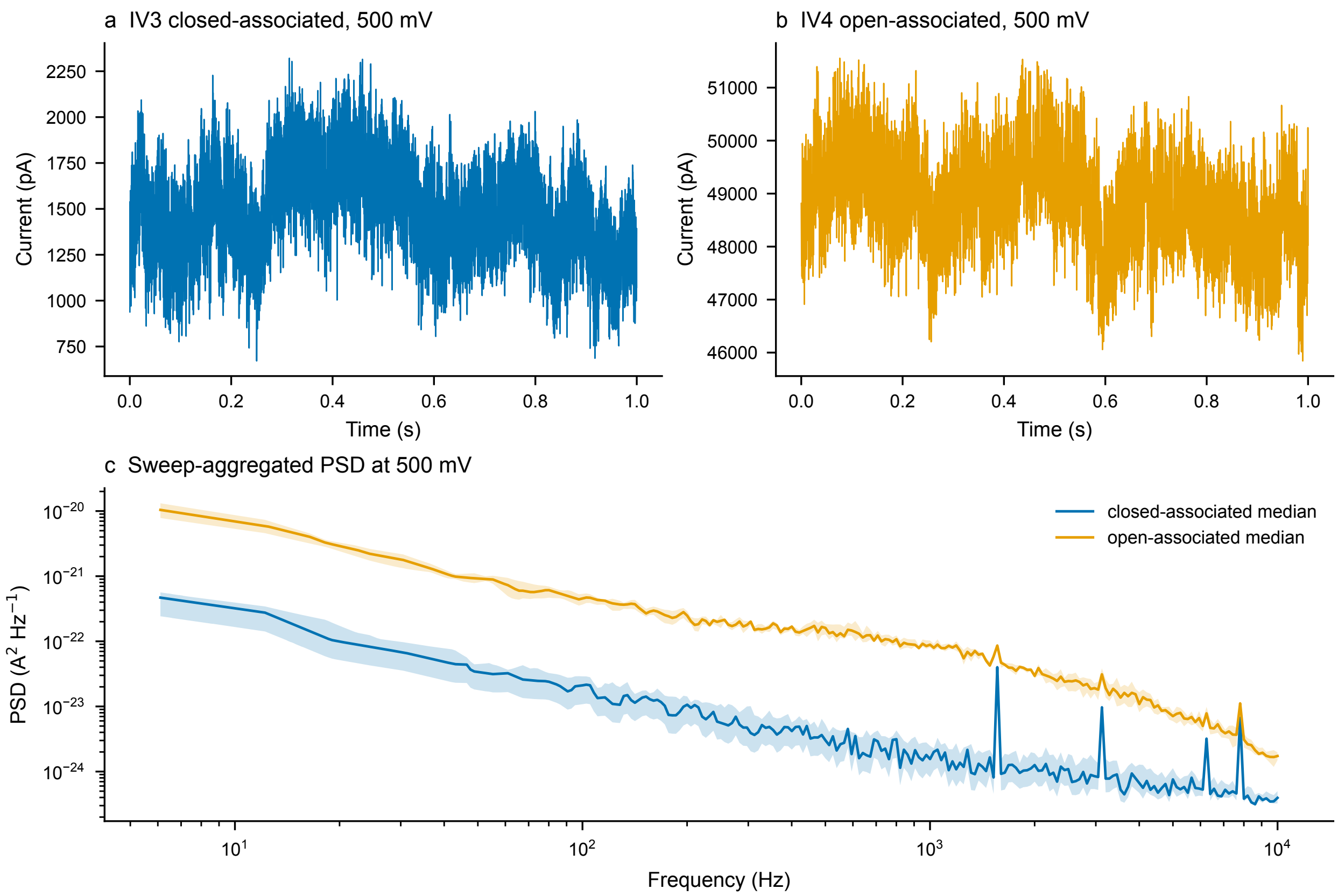

FIG. S1. Adjacent current traces and spectral comparison for the within-pore control. One-second portions of the first available +500 mV plateau from (a) IV3, the final closed-associated sweep, and (b) IV4, the first open-associated sweep. Panels (a) and (b) use different current-axis ranges so that fluctuations around each baseline remain visible. (c) Welch power spectral density (PSD) at +500 mV after median aggregation within each sweep. Solid curves and shaded regions denote the median and interquartile range across sweeps in each operational regime. PSD is reported in $A^2\ Hz^{-1}$. The regime assignment is electrical and does not independently validate microscopic wetting.

I. WITHIN-PORE, VOLTAGE-MATCHED CONTROL

The control data series comprises seven sequential voltage sweeps from one nominally 3 nm MoS_2 nanopore in 3 M KCl. IV1–IV3 were assigned the operational label "closed-associated," and IV4–IV7 were assigned "open-associated." These labels describe the recorded electrical regimes and do not constitute independent physical validation of microscopic wetting. All sweep-level comparisons used the same set of applied voltages.

II. SIGNAL-TRANSFORMATION SENSITIVITY ANALYSIS

Each valid 2 s current window was analyzed in three representations: the original signal, the mean-centered signal $I(t) - \overline{I}$, and the standardized signal $[I(t) - \overline{I}]/\sigma_I$. Here, σ_I denotes the population standard deviation within the window. The seven shape descriptors comprised three normalized PSD band fractions, the PSD slope from 10–1,000 Hz, lag-1 autocorrelation, skewness, and excess curtosis. For standardization, a window would have been excluded if its standard deviation was no larger than the greater of 10^{-30} A and $100\,\epsilon_{\mathrm{mach}} \max_t |I(t)|$, where ϵ_{mach} is the double-precision machine epsilon. None of the 239 valid windows met this exclusion criterion.

Descriptor values from the transformed signals were compared with their original-signal values using a relative tolerance of 10^{-8} and an absolute tolerance of 10^{-10}. All seven descriptors satisfied these tolerances under both transformations, giving 14 of 14 descriptor-transformation checks. This agreement verifies the intended numerical invariance of the implemented descriptors to baseline-current offset and absolute fluctuation scale. Such invariance is expected for these descriptor definitions and is not an independent validation of feature selection or microscopic wetting state.

For the regime comparison, window-level descriptor values were first aggregated by their median within each sweep–voltage combination, including values from repeated plateau files at the same voltage. Each descriptor was then centered separately within each matched nonzero voltage by its median across the seven sweeps and scaled by 1.4826 times its median absolute deviation. The population standard deviation was used when the robust scale was zero. The median across the ten matched nonzero voltages provided one summary per descriptor for each sweep.

The descriptive rank-bi-serial contrast was calculated as the mean sign of all pairwise differences between the four open-associated and three closed-associated sweep summaries, with ties contributing zero. Positive values indicate a tendency toward larger values in the open-associated sweeps; negative values indicate the opposite tendency. For the leave-one-sweep-out calculations, one sweep was omitted at a time and the contrast was recalculated using the remaining sweep summaries. The within-voltage scaling was retained from the complete seven-sweep calculation rather than refitted after each omission. The resulting contrasts are reported in Table S1. Six descriptors retained the same contrast direction in

TABLE S1. Stability of the voltage-matched shape-descriptor contrasts after removal of baseline-current offset and absolute fluctuation amplitude. Values are descriptive rank-biserial contrasts comparing open-associated with closed-associated sweep summaries, following within-condition aggregation, within-voltage standardization, and summarization across the ten matched nonzero voltages. The contrasts were identical across the three signal representations within numerical precision. LOO indicates whether the contrast direction remained unchanged in every leave-one-sweep-out calculation. These results do not represent independent-pore replication or predictive performance.

Descriptor	Original	Mean-centered	Standardized	LOO stable
PSD fraction, 1–100 Hz	1.00	1.00	1.00	Yes
PSD fraction, 100–1,000 Hz	1.00	1.00	1.00	Yes
PSD fraction, 1–10 kHz	-1.00	-1.00	-1.00	Yes
PSD slope, 10–1,000 Hz	-1.00	-1.00	-1.00	Yes
Lag-1 autocorrelation	1.00	1.00	1.00	Yes
Skewness	-0.25	-0.25	-0.25	No
Excess kurtosis	-1.00	-1.00	-1.00	Yes

every leave-one-sweep-out calculation; skewness was the exception. The windows and sweeps remain nested observations from one physical pore, not independent nanopore replicates.

III. DESCRIPTIVE TRANSITION-BOUNDARY SENSITIVITY

The seven sweeps in the control dataset were treated as an ordered sequence. All six possible boundaries between consecutive sweeps were evaluated using the seven shape descriptors described above. The input consisted of the sweep-level descriptor summaries obtained after within-condition aggregation, within-voltage robust standardization, and median summarization across the ten matched nonzero voltages. For the boundary comparison, each descriptor was additionally centered across the seven sweep summaries by its median and scaled by 1.4826 times its median absolute deviation. The population standard deviation was used when the robust scale was zero. This representation was held fixed when evaluating the six boundaries.

Let u_{sj} denote the resulting value for sweep s and descriptor j, with $s = 1, \ldots, 7$ and $j = 1, \ldots, 7$. For a boundary after sweep k, the adjacent-sweep separation was

$$J_k = \sqrt{\frac{1}{7}\sum_{j=1}^{7} (u_{k+1,j} - u_{k,j})^2}, \qquad k = 1, \ldots, 6.$$

The between-segment separation compared the descriptor-wise medians of the sweeps on either side:

$$D_k = \sqrt{\frac{1}{7}\sum_{j=1}^{7} \left[\text{median}_{s>k}(u_{sj}) - \text{median}_{s\leq k}(u_{sj})\right]^2}.$$

A balance factor reduced the weight of highly unequal segment sizes:

$$B_k = D_k \, \frac{2\sqrt{k(7-k)}}{7}.$$

Each descriptor contributed equally to these summaries; the descriptors were not assumed to be statistically independent. These measures provide a descriptive comparison of boundaries, not an inferential change-point test.

Both the adjacent-sweep separation and the balanced between-segment separation were largest at the interface of the segments IV2 and IV3. The operational boundary between the segments IV3 and IV4, ranked third under both measures (Table S2). Thus, the group-level descriptor contrasts did not uniquely identify the operational electrical transition. The boundary results do not negate the observed differences between the assigned groups. They limit their interpretation: those differences do not establish a unique transition point or demonstrate that wetting caused the changes across the sequential recordings.

IV. EXPERIMENTAL SETUP

The nanopore data analyzed in this work are obtained from the experiments performed in the Laboratory of Nanoscale Biology at the EPFL, Switzerland. The electrical measurements were performed using Ag/AgCl electrodes positioned in the cis and trans chambers of the PMMA flow cell. The electrodes were prepared by chlorinating Ag-wires, electrochemically in KCl solution by applying a positive potential forming a grayish AgCl coating. Approximately 1 cm Ag/AgCl electrodes are inserted into the two chambers of a flow-cell, with the cis-side electrode connected to ground and the trans-side electrode connected to the active terminal of the amplifier. The MoS_2 nanopore devices are fabricated as shown before[1]. Single-layer

TABLE S2. Descriptive separation at all six boundaries, denoted as IVi|IVj, between consecutive sweeps in the control data. J_k measures adjacent-sweep separation, and B_k measures balanced between-segment separation. Larger values indicate greater separation under the corresponding measure. Ranks were determined from unrounded values; numerical entries are rounded to two decimal places. All seven sweeps were recorded sequentially from one physical pore.

Boundary	Adjacent J_k	Balanced B_k	Rank under both measures
IV1|IV2	6.24	2.55	2
IV2|IV3	6.29	4.03	1
IV3|IV4	1.59	2.15	3
IV4|IV5	1.39	2.05	4
IV5|IV6	0.93	1.58	5
IV6|IV7	0.46	0.80	6

MoS_2 (1nm thickness) on a suspended SiN-membrane (20 nm thickness) separates the two chambers of the flow cell. The measurements are performed in a flow-cell with 1 M KCl, 10 mM Tris and 100 mM EDTA maintained at pH 7.5. The analyte solution is always filtered (20 nm) and degassed before measurements. Regarding the data acquisition, typically the recording is performed using Axopatch 200B (Axon Instruments, USA) at 10 kHz low-pass filtered and 100 kHz sampling rate for a few minutes until the events are observed[1,2]. When the pore was non-conductive, a transient reverse voltage (typically -100 to -500 mV) was applied to unblock the pore and continue measurement.

REFERENCES

[1]Graf, M., Lihter, M., Thakur, M., Georgiou, V., Topolancik, J., Ilic, B.R., Liu, K., Feng, J., Astier, Y. and Radenovic, A., 2019. Fabrication and practical applications of molybdenum disulfide nanopores. Nature protocols, 14(4), pp.1130-1168.

[2]Thakur, M., Macha, M., Chernev, A., Graf, M., Lihter, M., Deen, J., Tripathi, M., Kis, A. and Radenovic, A., 2020. Wafer-Scale Fabrication of Nanopore Devices for Single-Molecule DNA Biosensing using MoS2. Small Methods, 4(11), p.2000072.
